# Supplementary material for: Deep learning-based high-accuracy quantitation for lumbar intervertebral disc degeneration from MRI
Source: Nat Commun. 2022 Feb 11;13:841. doi: 10.1038/s41467-022-28387-5 (PMC8837609; doi:10.1038/s41467-022-28387-5)
Supplement: Supplementary file 1 — Supplementary Information [file 41467_2022_28387_MOESM1_ESM.pdf]

Supplement Table 1 Segmentation Performance on other MR images from different cites

Cites	Vertebral body		IVD		Lumbar spine	
	mDice	mIoU	mDice	mIoU	mDice	mIoU
Dongzhimen Hospital, Beijing University of CM	0.9567	0.9214	0.9198	0.8567	0.9193	0.8620
Guangdong Provincial Hospital of CM	0.9656	0.9498	0.9337	0.9046	0.9365	0.8888
Shenzhen Pingle Orthopedics Hospital	0.9654	0.9445	0.9334	0.8952	0.9269	0.8763
Longhua Hospital, Shanghai University of TCM *	0.9703	0.9425	0.9480	0.9019	0.9470	0.9035

* MR images from Longhua Hospital, Shanghai University of TCM were used to train the model as Data Set and to evaluate the accuracy of segmentation performance as control.

Supplement Table 2 Consistency analysis of intervertebral disc parameters calculated by MRI of different sizes

measurement	Intraclass Correlation [b]	
	ICC [a]	95%CI
ΔSI	.874***	(.840， .902)
DHI	.958***	(.943， .968)
HDR	.956***	(.886， .978)

*Two-way mixed effects model where people effects are random and measures effects are fixed. ICC, intraclass correlation coefficient; 95% CI, 95% confidence interval.

a. The estimator is the same, whether the interaction effect is present or not.

b. Type A intraclass correlation coefficients using an absolute agreement definition.

Supplement Table 3 Included patient demographic information from the four sites around China

Site	Number	Age(F/M)					
		20-29	30-39	40-49	50-59	60-69	70-89
Longhua Hospital, Shanghai University of TCM	433	32/21	52/51	49/45	34/35	53/39	12/10
Shenzhen Pingle Orthopedics Hospital	222	16/18	20/20	19/20	18/21	13/23	9/25
Guangdong Provincial Hospital of Chinese Medicine	246	19/24	20/15	23/17	22/17	18/15	22/34
Dongzhimen Hospital, Beijing University of Chinese Medicine	150	7/8	13/18	21/17	13/8	12/11	8/14
Total	1051	74/71	105/104	112/99	87/81	96/88	51/83

Supplement Table 4 Quantitative ranges of ΔSI according to the modified Pfirrmann Grade (1-8)

modified Pfirrmann Grade	1	2	3	4	5-8
Number	154	1130	1622	1315	1034
ΔSI (mean±SD)	121.97±9.96	95.34±7.20	72.34±7.81	44.63±8.49	20.60±9.28

Supplement Table 5 Mean and standard deviation (σ) of ΔSI of healthy discs for each lumbar level and each age

age		20-30	30-40	40-50	50-60	60-70	70-90
mean	L1L2	98.30	99.03	95.35	87.28	94.59	98.30
	L2L3	92.82	90.59	84.83	86.13	91.63	92.82
	L3L4	86.30	83.31	76.02	73.88	86.62	86.30
	L4L5	81.46	71.98	65.47	58.04	80.47	81.46
	L5S1	69.84	60.41	42.72	38.48	73.72	69.84
std	L1L2	17.92	15.50	18.55	21.70	25.09	17.92
	L2L3	18.99	16.03	23.19	19.94	22.35	18.99
	L3L4	20.47	22.88	17.73	20.80	20.37	20.47
	L4L5	20.61	21.42	21.77	29.57	20.14	20.61
	L5S1	28.56	23.66	16.16	35.90	22.14	28.56

Supplement Table 6 Mean and standard deviation (σ) of DH of healthy discs for each lumbar level and each gender and age

	age		20-30	30-40	40-50	50-60	60-70	70-90
female	mean	L1L2	12.28	12.60	12.89	13.45	13.55	12.26
		L2L3	13.81	14.57	14.81	15.28	14.61	15.46
		L3L4	15.36	16.03	16.36	15.93	15.77	15.16
		L4L5	16.48	17.15	16.57	16.48	16.34	15.71
		L5S1	15.05	15.75	16.18	16.91	16.35	15.03
	std	L1L2	1.71	2.13	1.53	2.22	1.48	2.79
		L2L3	1.54	2.37	1.57	1.89	1.62	4.26
		L3L4	1.44	1.90	1.37	1.69	1.66	1.98
		L4L5	1.88	1.96	1.64	2.22	1.80	1.74
		L5S1	2.13	2.42	2.68	2.47	3.54	3.17
male	mean	L1L2	13.30	14.11	14.54	15.40	14.70	13.90
		L2L3	15.02	16.24	16.15	17.13	16.68	15.93
		L3L4	16.56	17.51	17.49	17.26	17.14	16.29
		L4L5	17.34	17.89	18.00	17.71	17.16	15.84
		L5S1	15.37	15.96	16.21	16.89	17.68	15.87
	std	L1L2	1.47	2.30	3.04	4.75	3.94	2.10
		L2L3	2.42	2.78	2.11	4.60	3.75	4.07
		L3L4	1.73	2.14	1.80	2.67	2.34	1.61
		L4L5	2.02	2.30	2.09	2.15	2.34	2.77
		L5S1	2.23	2.38	2.44	2.46	2.56	3.44

Supplement Table 7 Mean and standard deviation (σ) of DHI of healthy discs for each lumbar level and each gender and age

	age		20-30	30-40	40-50	50-60	60-70	70-90
female	mean	L1L2	0.2640	0.2713	0.2793	0.2964	0.3057	0.2816
		L2L3	0.2929	0.3138	0.3162	0.3390	0.3295	0.3607
		L3L4	0.3311	0.3465	0.3530	0.3574	0.3623	0.3582
		L4L5	0.3639	0.3784	0.3673	0.3750	0.3699	0.3673
		L5S1	0.3337	0.3455	0.3586	0.3838	0.3742	0.3435
	std	L1L2	0.0435	0.0614	0.0399	0.0497	0.0462	0.0647
		L2L3	0.0388	0.0779	0.0404	0.0604	0.0479	0.1222
		L3L4	0.0411	0.0472	0.0342	0.0441	0.0537	0.0443
		L4L5	0.0502	0.0539	0.0420	0.0523	0.0507	0.0423
		L5S1	0.0578	0.0644	0.0671	0.0602	0.0903	0.0727
male	mean	L1L2	0.2801	0.2905	0.3063	0.3314	0.3178	0.3162
		L2L3	0.3156	0.3334	0.3368	0.3649	0.3674	0.3594
		L3L4	0.3539	0.3660	0.3694	0.3726	0.3757	0.3764
		L4L5	0.3843	0.3816	0.3861	0.3870	0.3751	0.3638
		L5S1	0.3375	0.3382	0.3493	0.3713	0.3811	0.3619
	std	L1L2	0.0360	0.0556	0.0715	0.1177	0.0906	0.0523
		L2L3	0.0556	0.0675	0.0490	0.1068	0.1224	0.1020
		L3L4	0.0410	0.0508	0.0455	0.0665	0.0540	0.0472
		L4L5	0.0415	0.0505	0.0517	0.0605	0.0519	0.0674
		L5S1	0.0491	0.0589	0.0597	0.0666	0.0657	0.0819

Supplement Table 8 Mean and standard deviation (σ) of HDR of healthy discs for each lumbar level and each gender and age

	age		20-30	30-40	40-50	50-60	60-70	70-90
female	mean	L1L2	0.2209	0.2218	0.2279	0.2310	0.2293	0.2024
		L2L3	0.2313	0.2488	0.2454	0.2494	0.2292	0.2483
		L3L4	0.2534	0.2629	0.2606	0.2448	0.2377	0.2234
		L4L5	0.2647	0.2688	0.2570	0.2434	0.2385	0.2308
		L5S1	0.2532	0.2584	0.2654	0.2683	0.2571	0.2296
	std	L1L2	0.0285	0.0358	0.0398	0.0338	0.0283	0.0484
		L2L3	0.0214	0.0963	0.0282	0.0914	0.0260	0.1108
		L3L4	0.0255	0.0377	0.0249	0.0326	0.0261	0.0344
		L4L5	0.0329	0.0345	0.0312	0.0361	0.0309	0.0289
		L5S1	0.0404	0.0444	0.0490	0.0373	0.0597	0.0522
male	mean	L1L2	0.2111	0.2213	0.2302	0.2686	0.2242	0.2202
		L2L3	0.2270	0.2399	0.2389	0.2518	0.2535	0.2646
		L3L4	0.2478	0.2552	0.2518	0.2447	0.2377	0.2324
		L4L5	0.2517	0.2554	0.2534	0.2475	0.2305	0.2203
		L5S1	0.2340	0.2430	0.2431	0.2475	0.2626	0.2414
	std	L1L2	0.0277	0.0529	0.0640	0.2721	0.0725	0.0344
		L2L3	0.0361	0.0431	0.0341	0.0774	0.1435	0.2789
		L3L4	0.0259	0.0246	0.0276	0.0460	0.0528	0.0241
		L4L5	0.0297	0.0361	0.0293	0.0533	0.0340	0.0424
		L5S1	0.0333	0.0310	0.0373	0.0410	0.0427	0.0520

Supplement Table 9 Imaging parameters for the MRI sequences in the 4 sites

Site	City	Strength of the Magnet	Company	Model	Coil
Longhua Hospital, Shanghai University of TCM<br><br>(Data Set A, B, C)	Shanghai	1.5-Tesla	SIEMENS	MAGNETOM Aera XJ	18-channel Spine Tim 4G coil
Guangdong Provincial Hospital of Chinese Medicine<br><br>(Data Set C)	Guangzhou	3-Tesla	SIEMENS	TIM Systems	32-channel Spine Tim coil
Shenzhen Pingle Orthopedics Hospital<br><br>(Data Set C)	Shenzhen	1.5-Tesla	SIEMENS	MAGNETOM Essenza	8-channel quadrature body coil
Dongzhimen Hospital, Beijing University of Chinese Medicine<br><br>(Data Set C)	Beijing	1.5-Tesla	SIEMENS	MAGNETOM Amira	24-channel quadrature body coil

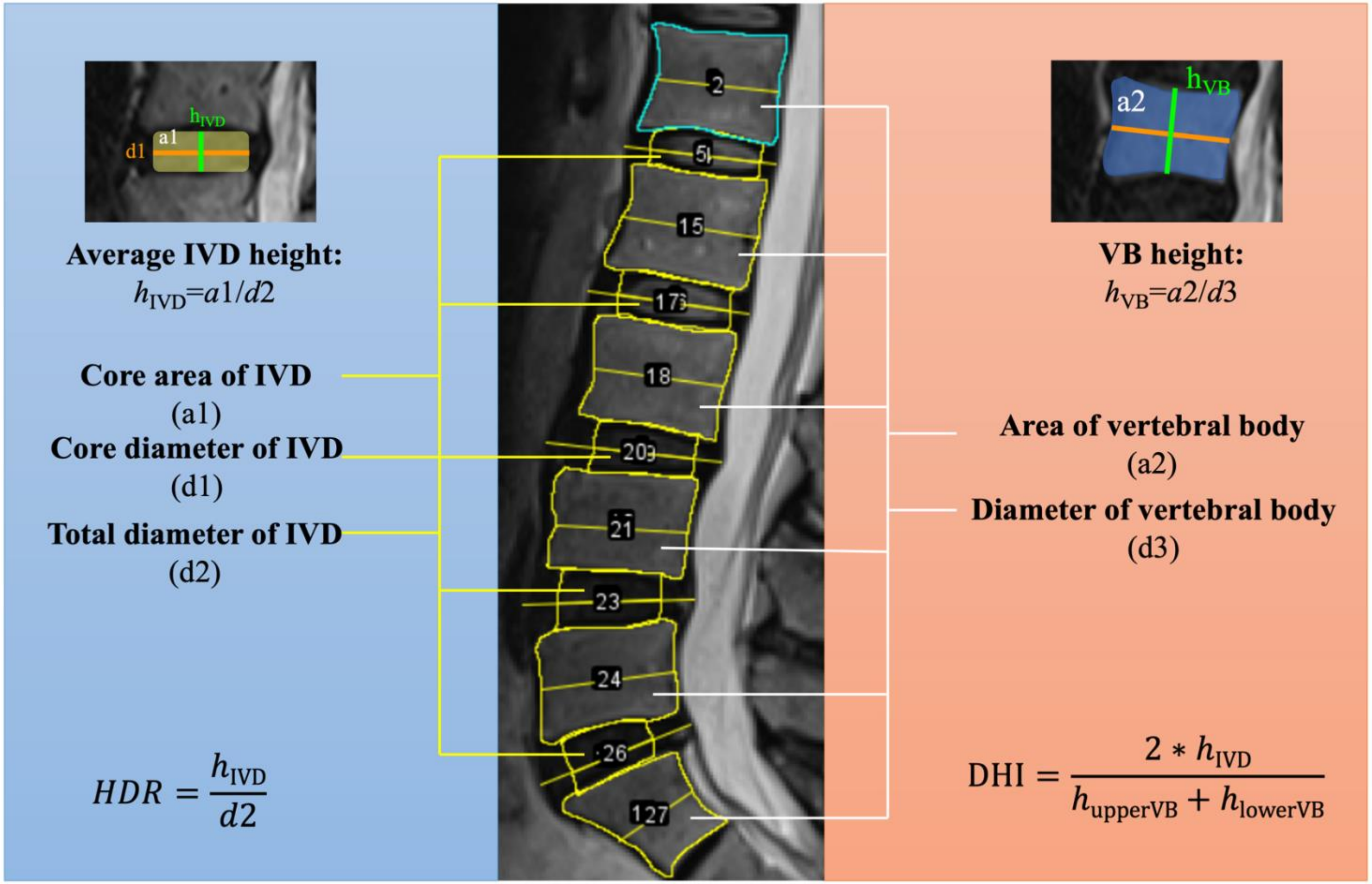

Supplement Figure 1 Scheme of manual measurement for IVD related areas.

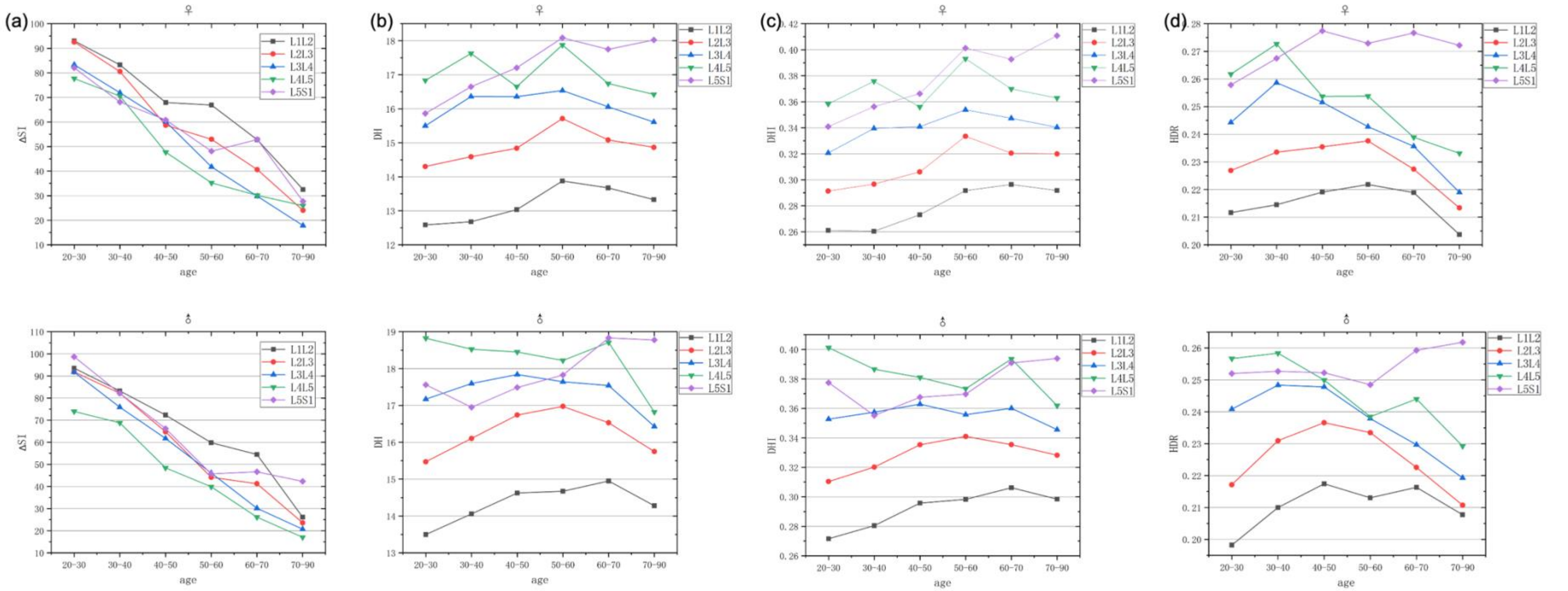

Supplement Figure 2 baseline characteristics of IVD parameters in a larger population.

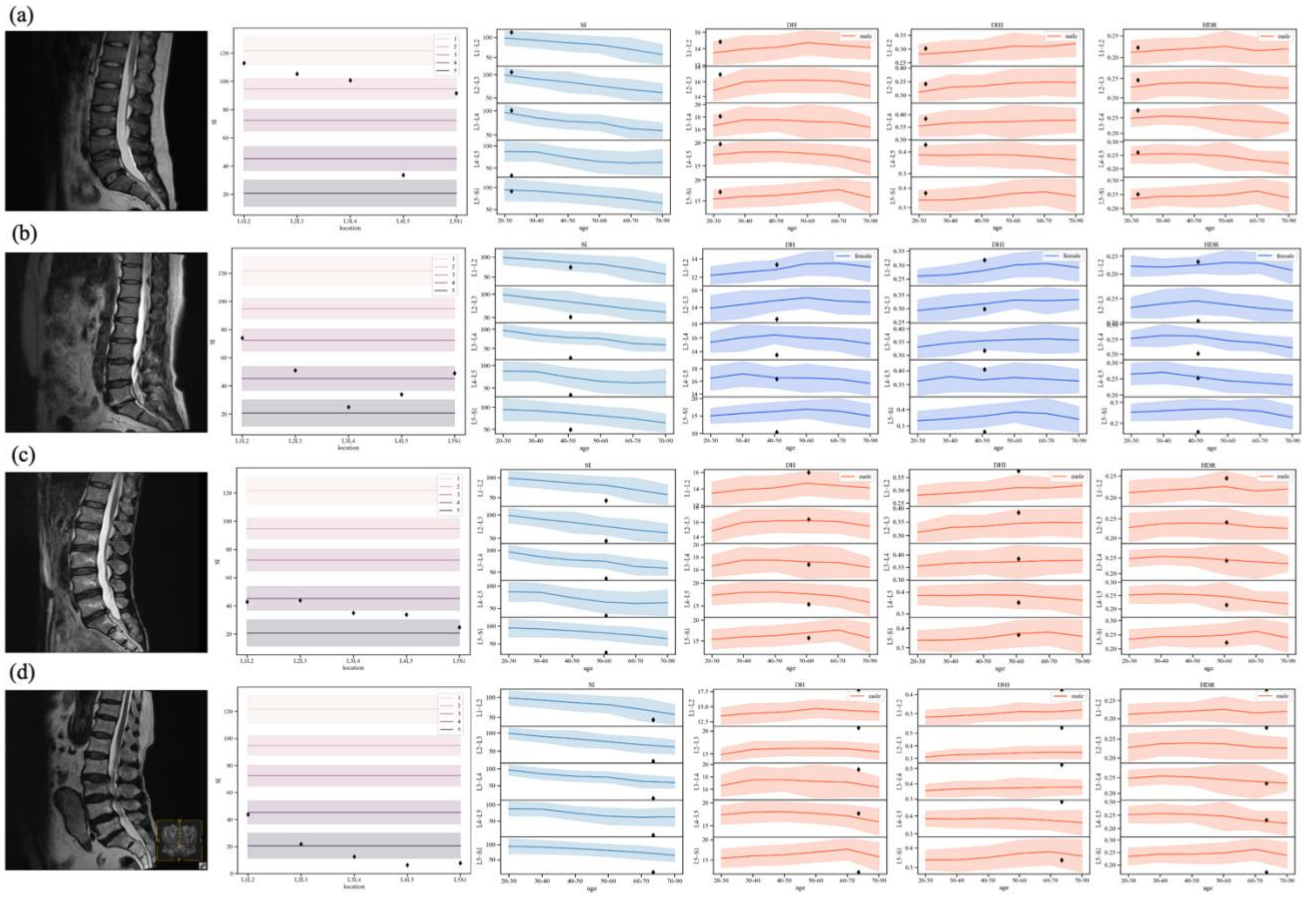

Supplement Figure 3 Quantitative analysis results of typical cases. (a) 23-year-old male; (b) 49-year-old female; (c) 63-year-old male; (d) 81-year-old male.

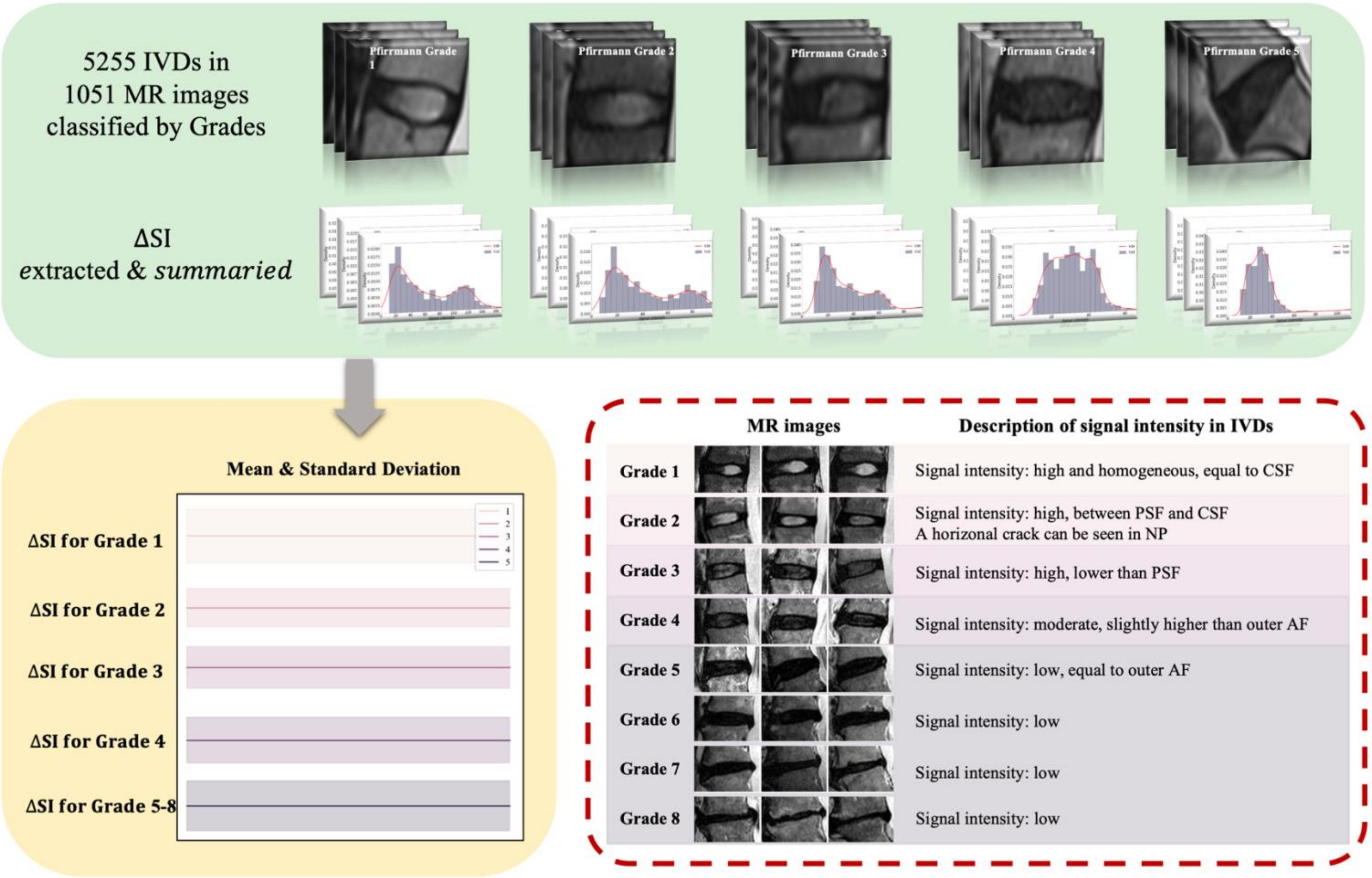

Supplement Figure 4 Scheme of IVD degeneration quantitation
