# Supplementary material for: Deep learning-based high-accuracy quantitation for lumbar intervertebral disc degeneration from MRI
Source: Nat Commun. 2022 Feb 11;13:841. doi: 10.1038/s41467-022-28387-5 (PMC8837609; doi:10.1038/s41467-022-28387-5)
Supplement: Supplementary file 2 — Description of Additional Supplementary Files [file 41467_2022_28387_MOESM2_ESM.pdf]

Title: Supplementary Video 1

Description: BianqueNet demo in IVD degeneration quantitation

The signal intensity quantitation result is divided into three parts: (1) signal intensity histogram of each IVD region; (2) the difference between the peak value of the disc signal intensity; (3) degeneration grade determination according to IVD signal intensity.

The geometric quantitation result is mainly divided into two parts: (1) DHI and HDR values of IVDs in each segment; (2) scatter diagram of each segment of IVD relative to the population standard.
